# Cryogenic propellant management in space: open challenges and perspectives

**DOI:** 10.1038/s41526-024-00377-5

**Published:** 2024-03-20

**Authors:** Alessia Simonini, Michael Dreyer, Annafederica Urbano, Francesco Sanfedino, Takehiro Himeno, Philipp Behruzi, Marc Avila, Jorge Pinho, Laura Peveroni, Jean-Baptiste Gouriet

**Affiliations:** 1 Liquid and Solid Propellant Research Expert Group, von Karman Institute for Fluid Dynamics, 72, chaussée de Waterloo, Rhode-Saint-Genèse, 1640 Belgium; 2 Center of Applied Space Technology and Microgravity (ZARM), University of Bremen, Am Fallturm 2, Bremen, 28359 Germany; 3 Fédération ENAC ISAE-SUPAERO ONERA, Université de Toulouse, 10 avenue Edouard Belin, Toulouse, BP 54032 - 31055 France; 4 Department of Aeronautics and Astronautics, The University of Tokyo, 7-3-1, Hongo, Bunkyo-ku, Tokyo, 138656 Japan; 5 JTLF, ArianeGroup GmbH, 1 Airbus-Allee, Bremen, 28199 Germany

**Keywords:** Aerospace engineering, Fluid dynamics

## Abstract

This paper presents open challenges and perspectives of propellant management for crewed deep space exploration. The most promising propellants are liquid hydrogen and liquid methane, together with liquid oxygen as an oxidizer. These fluids remain liquid only at cryogenic conditions, that is, at temperatures lower than 120 K. To extend the duration of space exploration missions, or even to enable them, the storage and refueling from a cryogenic on-orbit depot is necessary. We review reference missions, architectures, and technology demonstrators and explain the main operations that are considered as enablers for cryogenic storage and transfer. We summarize the state of the art for each of them, showing that many gaps in physical knowledge still need to be filled. This paper is based on recommendations originally proposed in a White Paper for ESA’s SciSpacE strategy.

## Introduction

Since the first crewed mission reached the surface of the moon more than 50 years ago, many nations strive for more open and broader access to space. The research and development to enable and improve space accessibility by robotic machines and human beings led to a discovery boost in several fields. Physical and biological sciences have had a new ground of exploration, but new technologies have also enabled a better life on Earth^1^. After more than 50 years, research and development is still needed to push the boundaries of space exploration. Human space endeavors beyond low Earth orbit (LEO) are now the new target^2–4^. Propulsion is an important subsystem of a spacecraft or launcher, and propellant is by far its largest mass fraction. For instance, ~98.5% of the Saturn V launch mass was propellant and propulsion systems^5^.

Many national space agencies are working to enable deep space exploration. One example is NASA’s exploration program, which outlines the basic requirements for future exploration missions^3^. On 14.10.2020, one of NASA’s tipping point selection was: “Cryogenic Fluid Management Technology Demonstration: NASA and industry partners have developed and tested numerous technologies to enable long-term cryogenic fluid management, which is essential for establishing a sustainable presence on the Moon and enabling crewed missions to Mars. Implementation of the technologies in operational missions requires further maturation through in-space demonstrations.” The awarded companies are Eta Space of Meritt Island, Florida, Lockheed Martin of Littleton, Colorado, SpaceX of Hawthorne, California, and United Launch Alliance (ULA) of Centennial, Colorado.

In the last two decades, several propulsion systems have been proposed and analysed for deep space exploration. The most promising ones are those fully based on nuclear thermal power (requiring liquid hydrogen) and on nuclear electric power plus cryogenic chemical propulsion for large velocity change maneuvers^3,6,7^. The studies^6^ and^7^ show that large amounts of cryogenic fuels need to be stored in space and transferred between spacecraft. For this reason, we focus this review paper on physical problems which are encountered in cryogenic systems only^8^. Cryogenic fuels (propellants, i.e., hydrogen, methane, and oxidizer, i.e., oxygen) have several advantages: they provide a high specific impulse, are non-toxic, and can be produced in situ (In Situ Resource Utilization - ISRU), i.e., on the surface of the Moon or Mars^9^. The enabling capabilities for cryogenic propellants are the long-term storage in space and on planets, and the transfer between depots and spacecraft. Depots will be launched empty or partly filled, and need to be refilled in space. A series of documents that explain the actual limitation and the current interest in cryogenic propellant management physics can be found in the references ^10,11^.

This review is organized in two parts. Firstly, the application perspectives are presented, summarizing the conditions of a typical reference mission and the associated architecture. This part is used to identify the requirements in terms of the size and working conditions of a possible tank enabling human deep space exploration. Then, the most relevant technological demonstrators are discussed as background knowledge. The most common phenomena occurring in a typical architecture are illustrated together with gaps in physical knowledge associated with cryogenic propellant management.

## Application perspectives and background

### Reference missions and architectures

A successful exploration mission requires spacecraft to change their velocity (also called △*V* maneuvers) in order to follow precise trajectories. Velocity change requirements are derived from orbital and spaceflight mechanics, involving the balance of propulsive, gravitational, and aerodynamic forces when entering the atmosphere of planets. The propulsive forces are produced by the spacecraft engine. Propellants are grouped into storable and cryogenic propellants. The main difference is the achievable specific impulse *I*_sp_ or thrust per unit mass flow rate, which is defined as the ratio between the thrust and the product of mass flow rate and the gravitational acceleration constant. Storable propellants (hydrazine, monomethylhydrazine, and nitrogen tetroxide) have a lower specific impulse, but they remain liquid at ambient conditions and do not need cooling. On the other side, cryogenics are in a gaseous phase at ambient conditions, because they are characterized by very low saturation temperatures at normal pressure, 101,325 Pa (20 K for hydrogen, 112 K for methane). As a consequence, they need to be cooled down in order to be stored in a liquid phase and require good insulation.

Design reference missions (DRM’s) are required to compute the necessary velocity changes. The Artemis program is the current NASA project to return humans to the moon’s surface, with the purpose to stay and to prepare for human Mars exploration missions. The first Artemis uncrewed mission around the Moon was completed in December 2022 with the first flight of the Space Launch System (SLS) and the second flight of the Orion Crew Exploration Vehicle (CEV) with the European Service Module (ESM) (see nasa.gov/specials/artemis).

A crewed Mars mission requires larger velocity changes and shall be used here as an example^12^. Conjunction and opposition class missions were taken by Oleson et al. in 2021^6^ for a concept of operations (see their page 24). The baseline is a nuclear electric propulsion (NEP)—a chemical vehicle with liquid methane and liquid oxygen for high-thrust maneuvers. Typical mission milestones are:

1. Several heavy launches from Earth to Low Earth orbit (LEO)
2. Rendezvous and docking, assembly in LEO
3. Transfer to trans Lunar orbit (TLO)
4. Rendezvous and docking with habitat
5. Heavy launch from Earth to TLO with CEV and crew transfer to habitat
6. Trans Mars injection (TMI)
7. Injection into Mars orbit
8. Descent to Mars surface
9. Surface mission (30 days)
10. Ascent to Mars orbit
11. Trans Earth injection (TEI)
12. Trans Lunar orbit and crew transfer to the CEV
13. Return to Earth surface

This concept of operations requires a tanker spacecraft to fuel the chemical stage in Earth orbit.

Other scenarios (see ref. ^7^) require nuclear thermal propulsion with liquid hydrogen as propellant, heated by the nuclear reactor. This does not require an oxidizer and produces the highest specific impulse. More information on possible mission scenarios, road maps, and technological challenges are given by refs. ^10,13–15^.

In this review, we follow the rationale of Hartwig^8^ for propellant depots in orbit. Without the ability to fuel and/or refuel spacecrafts in orbit, all the propellant has to be taken from Earth to reach the destination and return to Earth. This traditional method requires a heavy launch vehicle, such as the Space Launch System (SLS), with a payload mass of 95 t to LEO and 27 t for trans Lunar injection. This will not be sufficient to send humans beyond the Moon. A possible solution is to use a propellant depot.

A propellant depot is defined as an orbiting propellant storage vessel that can host fuels for up to several years^7^. The depot shall be launched and brought to its final orbit in an empty or partially filled state, since its wet mass might exceed the capacities of available launchers. Propellant transfer from a tanker to the depot and from the depot to an exploration spacecraft is required. Depots have multiple advantages:

1. The dry mass of an exploration payload, launched from the surface of the Earth, may be larger, because it will be fueled in space.
2. Commercial launch services can be used to supply and re-supply the depot.
3. The depot could be used to fill or re-fill the exploration spacecraft.
4. Depot technologies could be used to enhance planetary and Earth sciences^16^.

Cryogenic fluid management (CFM) technologies are required to enable all necessary steps, such as draining, chill down, transfer, and filling in both directions. In Table 1, rough order of magnitude numbers from different studies on propellant depots in space are summarized.

**Table 1:** Rough order of magnitude parameters for propellant depots in space

Depot type	dual fluid, 6 to 8 tanks	^17^
Capacity	50 t per tank	
Diameter	5 m	
Length	10 m to 20 m	
Fluids	hydrogen, methane, oxygen	^11,114^
Storage time	6 month to 12 month	^20^
Acceleration	From microgravity to high-g	^8,17^
Pressure	100 kPa to 350 kPa	^8,11^
Conditioning	Autogeneous pressurization	^8,11,13,115^
Heat flux	50 W m^2^ to 100 W m^2^	^26,116^
Transfer rate	0.15 kg s^1^ to 4.4 kg s^1^	^117,118^
Wall material	Aluminum	^25^

The data have been compiled from different sources.

An important consideration is the location of the depot. First of all, a concept of operations (CONOPS) is required. Probable locations of the depot (or depots) are:

1. low Earth orbit (LEO), height of orbit above surface 160 km to 1000 km, time of orbit 90 min
2. geosynchronous orbit (GEO), height of orbit above surface 35,786 km, time of orbit 24 h
3. Earth-Moon Lagrange 1 orbit (EML1), distance of the orbit from the center of the Earth 326,400 km (85% of the Earth-Moon distance of 384,400 km), time of orbit 29.5 d
4. Earth-Moon Lagrange 2 orbit (EML2), distance of the orbit from the center of the Earth 448,900 km, i.e., 60,000 km beyond the Moon, time of orbit 29.5 d

Some features are the △*V* requirements to reach the depot and to keep the depot where it belongs, the thermal loads, the storage duration, and the possible problems associated with radio interference and space. The most important quantity is the boil-off loss. This is the mass of liquid propellant which is converted into a gas due to the incoming heat fluxes.

Possible architectures for such orbital propellant depots have been considered by Gaebler et al. in their 2009 paper^17^. An example of an orbital depot with multiple propellant tanks is shown in Fig. 3 in ref. ^17^. It should be capable of storing 330 t of propellant in space. Each tank measures 11 m in length and 5 m in diameter. A total of 8 tanks are considered. A reusable transfer vehicle (RTV) will perform transits between the low Earth orbit (LEO) of the depot and a low Lunar orbit (LLO). An example of such a vehicle is shown in ref. ^17^ in figure 4.

In their 2010 paper, ref. ^18^ advocate for a depot-based space transportation architecture. The concept consists of a very basic depot in LEO and subsequent EML1 and EML2 depots. The EML2 location seems to be the best due to the thermal, micrometeoroid, and atomic oxygen environment. Propellant stocked at EML2 is nearly at Earth escape energy. Transfer between LEO and EML2 requires a △*V* = 3.2 kms^1^. The depot development begins with a test-bed, continues with Centaur-based structures, and reaches a 120 t dual propellant containment which has an annual flow through of 300 t. Nevertheless, gas hydrogen created due to boil-off can be reused as well as monopropellant for station keeping and other purposes. The depots could serve multiple purposes, such as Moon exploration and crewed Mars missions, and be refueled from the Moon surface once in situ resources utilization (ISRU) capacities are available.

In their 2011 paper, Smithermann and Woodcock^5^ provide detailed information for eight reference missions:

1. Geo-synchronous orbit satellite servicing
2. Crew transfer vehicle (CTV) between LEO depot and EML1 depot
3. Lunar lander
4. Reusable upper stage (RUS) cargo
5. Earth-Sun Lagrange point 2 mission using the RUS, CTV, and a Deep Space Habitat (DSH)
6. Mars orbital depot delivery
7. Crew mission to Mars orbit
8. Semi-cycler crew mission to Mars orbit

Their study presents extended tables for these reference missions: △*V*, specific impulse, mass ratio, propellant mass, hardware mass, and remaining mass.

In 2016, Perrin and Casler^19^ sought to determine the optimum architecture for a fuel depot supplied from lunar assets. Their study concluded that EML1 is the best location for an orbiting depot. The design reference missions are commercial satellite servicing mission (CSS), Mars cargo mission (MC), and propellant delivery mission. △*V* requirements and time-of-flight calculations are presented based on two-body orbital mechanics. Depot locations are LEO, GEO, and EML1. The boil-off consideration requires knowledge of the heat fluxes at different locations. Some numbers are compiled in Table 2.

**Table 2:** Summary of features for different depot locations from^19^

Location	Total propellant mass kg	Heat flux Wm^2^	Boil-off rate kg h^1^	chill-down mass kg/transfer
LEO	434696	2162	0.02 to 0.20	5.6
GEO	212696	1388	0.01 to 0.13	5.6
EML1	204871	1367	0.01 to 0.12	5.6

The total propellant mass is based on fueling the commercial satellite servicing mission (CSS) vehicle and the Mars cargo mission (MC) vehicle every 6 months. The heat flux comes from the sun and the albedo of the Earth. The boil-off rates and chill-down masses are for hydrogen only.

A typical storage time of fuels will be of the order of months^20^ or even years^6^. The tank itself might undergo different kinds of accelerations during a complete mission, e.g. launch, ballistic phase, rendezvous, and station keeping. Consequently, the fluids within the tanks will behave differently depending on the acceleration environment^21^.

The operative pressure in the tanks will be in the range of 100 to 350 kPa^8,11^. Prior to the transfer, the liquid inside the tank needs to be in an appropriate thermodynamic condition. A thermodynamic equilibrium will be achieved in long storage phases, bringing the vapor and the liquid to saturation, even with a pressure rise due to incoming heat fluxes. A liquid at saturation will immediately start to evaporate, if the pressure is lowered in the transfer line and in the receiver tank. The conditioning of propellants prior to the transfer to the engines is currently performed by depressurization, followed by pressurization^22^. The depressurization sacrifices some liquid, which evaporates, and cools down the remaining bulk. The gas can be used for other purposes. Pressurization can be achieved in two ways: heterogeneous pressurization with a non-condensable gas, or autogeneous pressurization with the same species. A certain degree of subcooling can be kept for a limited period of time, and enable the transfer of liquid without vaporization due to pool or flow cavitation.

Typical heat fluxes towards the tank range from 1 W m^2^ with multi-layer insulations (MLI) to 100 W m^2^ without MLI^23,24^. The heat flux leads to the increase of the internal energy of the fluids (vapor and liquid) and may cause pool boiling at inner structures, either in a subcooled or saturated state. The surface structure of the wetted surface of the tank plays an important role in the creation of active nucleation sites, at which boiling will occur. Upper stage tanks are built from aluminum^25^. The topology and the chemical composition of the internal solid surfaces decide the wall superheat, which is required to trigger the generation of a bubble germ. Heat fluxes do not enter evenly, as outlined by ref. ^26^.

**Fig. 1:**
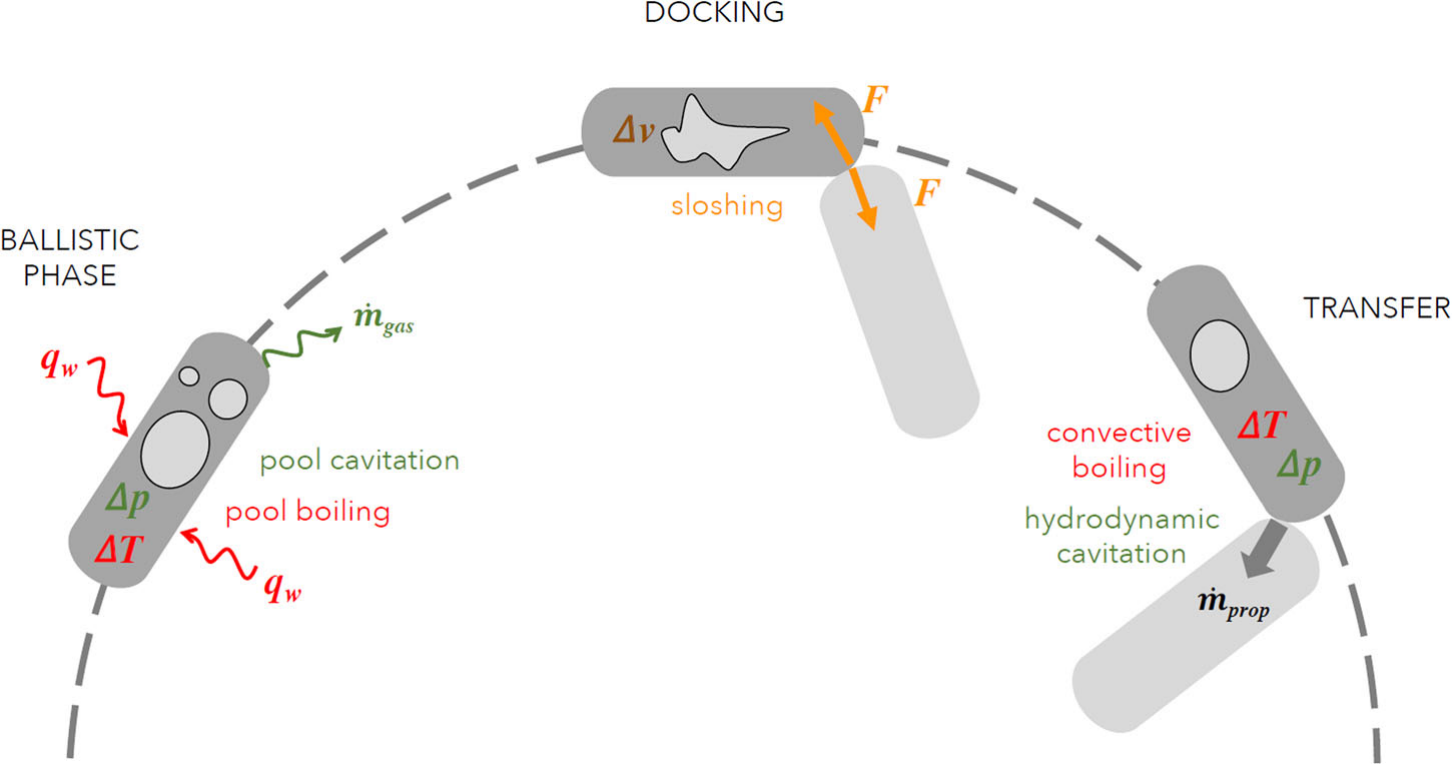
Scheme of operations for an orbiting depot and associated physical phenomena. The three operations represented are ballistic phase, docking and transfer. For each phase the most typical physical phenomena occurring is represented: in green the phenomena dominated by a variation in pressure, in red those dominated by a variation in temperature and in yellow those dominated by a variation of velocity.

The typical operation phases of a depot are storage, conditioning, maneuvers, and transfer. Several dominating physical phenomena can be associated with each phase. The understanding and the capability to model and simulate these phenomena is necessary to enable and improve technical solutions. We have compiled the main operation phases and their effects on the fluid systems in Table 3. In addition, a simple sketch shown in Fig. 1 visualizes the most important features of a propellant depot in space. The paper is organized along these lines, and more details will be reported in the following sections, based on the current state of the art.

**Table 3:** Operation phases (OP), effects, and associated physical phenomena (APP)

OP	Effect	APP
Storage	Near saturation conditions	Boiling caused
		by superheated wall
		microzone physics
Conditioning	Pressure variations	Bulk cavitation
		Bubble growth,
		shrinkage, and collapse
Maneuvers	Dynamic interaction between	Gas/liquid interface position
	fluid and structure	Liquid sloshing
Transfer	Pressure drop	Gas/liquid interface position
	in tanks and lines	Phase separation
		Cavitation
	Line chill down	Flow boiling
		Heat transfer modes
	Interaction supply/receiver	Dynamic models
	tank	liquid transfer
	Receiver tank filling	Interface stability
		Vented or no-vent filling

### Technological demonstrator

The technological demonstrators presented in this review mainly have the purpose to demonstrate that a system or a subsystem works in relevant conditions. NASA has considered that, among others, propulsion systems utilizing cryogenics are necessary for achieving future long-duration missions beyond the moon^11,13^. Launchers with cryogenic propellants have been used for decades with the mission of sending satellites into low Earth orbit or to re-supply the international space station. For this reason, technologies exist for cryogenic fluid management and storage, which, nevertheless, was intended to allow its use for a few hours: the state of the art for cryogenic storage is 14 h^20^. In order to enable human missions to Mars, the requirement is to store cryogenic fuels for months or possibly years^20^. Moreover, cryogenic liquids are highly susceptible to phase change resulting from even minute changes in pressure and temperature. Since cryogenic liquids tend to vaporize at common testing conditions, the measurement of their flow rate is compromised by the difficulty in quantifying the fluid quality (Percentage of gas mass with regard to the total mass). In the field of propulsion, this can present itself as a serious impediment to reliable engine performance, as predictable and repeatable propellant flow rates are required to ensure proper combustion and resulting thrust^27^.

Many propellant depot technological demonstrators have been attempted without reaching space^28–31^. In some cases, the technology reached space, but the failure of some systems did not allow to retrieve the expected results. This is the case of Sloshsat-Flevo^32–34^, a spacecraft for the experimental study of liquid dynamics and liquid management problems in space, launched in GTO in February 2005. The tank instrumentation did not respond after being on-orbit, but some qualitative results about the dynamic response of the sloshing fluid mass could be retrieved from the satellite control instrumentation.

The study of cryogenic management in microgravity has an impact on different technologies, such as Propellant Management Devices (PMD), Zero Boil-Off tanks (ZBO), Vapor Cooling Systems (VCS), Thermodynamic Vent Systems (TVS), Filling Operations (FO), Propellant Chill-Down (PTCD), Transfer Operations (TO), and Heterogeneous/Autogenous Pressurization (HP/AP)^20^. Several representative technology demonstrators for space propellant systems reported in the literature are summarized in Table 4.

**Table 4:** Technology demonstrators for cryogenic tanks

Reference	Investigation	Technol.	Gravity Conditions	Fluids	Highlights
Early tests^35–37^	Liquid hydrogen behavior in compensated gravity	PMD	Suborbital flights	Hydrogen	First series of hydrogen tests on sounding rocket experiment, 4 flights.
Flachbart et al.^38^	Effect of helium pressurant on TVS performance with liquid hydrogen	TVS HP	Ground	Liquid hydrogen, gaseous helium	Thermal energy removal from the ullage by spray.
Mustafi et al.^39^	Design of an isobaric subcooling system	PTCD	Ground	Hydrogen, oxygen	Subcool the liquid prior to launch. Conceptual design proposed. Not an official demonstrator yet.
Wang et al.^40^	Comparison of filling pressure and temperature in vented and non-vented cryogenic tanks	TVS FO	Ground	Nitrogen	Thermodynamic state of vented and no-vented fills is different, so correlation should be developed.
Flachbart et al.^41^	Liquid hydrogen tank rapid chill and fill testing	PTCD FO	Ground	Hydrogen	Tank structure chill-down process was slow. No-vent fill operation did not succeed.
Behruzi et al.^42^	PMDs usable for upper stages tested with liquid nitrogen	PMD	Suborbital flight	Nitrogen	Results are applicable to the real scale (size and fluids) PMDs based on the used scaling laws.
Leudiere et al.^43^	Liquid hydrogen behavior in compensated gravity	PMD	Suborbital flights	Hydrogen	First European hydrogen experiment in a sounding rocket, conducted by industry.
Plachta et al.^44^	Zero Boil-Off test for oxygen tank	ZBO	Ground	Oxygen	Active cooling system for oxygen eliminated boil-off and robust controlled tank pressure.
Sarae et al.^45^, Kinefuchi et al.^46^	Chill-down process in a complex channel	PTCD	Suborbital Flight	LN2	The transition of flow regimes from gas-liquid two-phase flow to liquid mono-phase flow was visualized.
Kassemi et al.^47^	Comparison of CFD and experimental data from 3 different datasets of self-pressurization	HP	Ground, ISS, Space Shuttle	Hydrogen, PnP, Freon-113	Only experimental controlled and known boundary conditions allow a good agreement with experiments and CFD of two-phase systems.
Breon et al.^48^	Storage and transfer of a cryogenic fuel on orbit	ZBO TO HP/AP TVS	Ground, ISS	Nitrogen, Argon, Methane	4 months Zero Boil-Off methane storage by a cryocooler. Problem during on-orbit venting expelled all methane prior to transfer demonstration.

The first documented experiments of propellant management devices operating with liquid hydrogen in a compensated gravity environment were performed in 1962^35–37^. Flachbart et al.^38^ evaluate the effects of helium pressurant on the performance of a spray-bar TVS to demonstrate the capability of pressure control for liquid hydrogen. A thermodynamic cryogen subcooler has been proposed by ref. ^39^, by removing energy from the cryogenic propellant through isobaric subcooling of the cryogen below its normal boiling point prior to launch. This simple technique can extend the operational life (factor of 2) of a spacecraft or an orbital cryogenic depot for months with minimal mass penalty. A comparison between venting and non-venting cryogenic storage tanks has been performed by ref. ^40^ for a 180 L cryogenic cylinder with vertical injection at on-ground conditions. They analysed the fluid and wall temperature distribution for both configurations and reported that two pressure regions occur during vented filling: pressure rise and gradual decrease. The observed regions during no-vented fill are pressure rise, gradual balance, and sharp pressure increase. Liquid hydrogen tank rapid chill and fill of a flight volume tank testing were performed on the ground by ref. ^41^. They found that the tank structure chill-down process was slow due to flow boiling developed at the walls, which changed the heat transfer coefficient. No-vented fill operation did not succeed. Nevertheless, as buoyancy inhibits the vapor film formation, the authors suggest that on-orbit filling would promote the creation of the vapor layer at the wall, increasing the efficiency of the cool-down process. Propellant management devices (PMD) for cryogenic liquids were tested in compensated gravity conditions (TEXUS 48 Sounding Rocket) using liquid nitrogen by Behruzi et al.^42^. Different operations were tested: draining, refilling, heating, and depressurization. A successful operation could be demonstrated. The first liquid hydrogen microgravity experiment performed in Europe was presented by ref. ^43^. They embarked on a module containing a cryogenic cell on a sounding rocket at SSC with two tanks of 2 L and 20 L of liquid/gas hydrogen, fully instrumented by temperature, pressure, and level sensors. High-speed cameras were placed to observe the behavior inside the tanks. The main objective for the mission was to study the behavior of liquid hydrogen under controlled gravity conditions, created by a cold gas thrust module. This experiment was conducted to support the development of liquid propellant management systems for the Ariane launcher. The use of active cooling systems such as cryocoolers eliminates boil-off for tanks filled with liquid oxygen, as demonstrated by ref. ^44^. Nevertheless, significant work still needs to be done on cryocooler integration for on-orbit tanks, especially for liquid hydrogen. The boiling flow of cryogenic nitrogen in complicated channels under low-gravity condition was realized with the sounding rocket’s suborbital ballistic flight by JAXA and the University of Tokyo^45,46^. The transition of flow regimes from gas-liquid two-phase flow to liquid mono-phase flow was visualized. Compared with the corresponding ground test, it was confirmed that the two-phase flow in the complex channel could wet the heat transfer surfaces more easily due to the absence of gravity, and that a more uniform chill-down effect could been obtained. Kassemi et al.^47^ proposed an experimental and CFD comparison for three different experimental databases on self-pressurization (AP) at Earth gravity and microgravity. The three databases come from the ZBOT experiment at normal and compensated gravity conditions (ISS) performed with perfluoro-n-pentane (PnP), the ground K-site tank experiment performed with liquid hydrogen, and the TPCE (Tank Pressure Control Experiment) experiment on the Space Shuttle, performed with Freon-113. The authors conclude that when measurements are taken under tight experimental control and known boundary conditions, the agreement with two-phase CFD results is good (for both large and small Bond number regimes), even though these models often use semi-empirical coefficients, such as the accommodation coefficient. A quite unique technological demonstrator for the storage and transfer of liquid methane was developed by ref. ^48^: the Robotic Refueling Mission 3 (RRM3). This mission extends RRM1 and RRM2, which demonstrated satellite refueling operations in a platform installed outside the ISS. As cryogenics cannot be handled inside the ISS for safety reasons, RRM3 is operated outside the ISS. Several operations have been demonstrated, among which was a four months zero boil-off methane storage by means of a cryocooler. Unfortunately, a problem during on-orbit venting expelled all methane prior to the transfer demonstration.

## Gaps in physical knowledge

In the previous section, we have seen what the perspectives of deep space exploration are and what is achieved with technological demonstrator. In this section, we summarize the state of the art on gaps in physical knowledge identified as enablers for the required operations (see Table 3). The demonstrators attempt to advance technologies, based on engineering assumptions and correlations. However, the data collected from technology demonstrators are difficult to be exploited for physical modeling necessary to master complex thermo-fluid-dynamic systems. Simplified experiments under controlled boundary conditions are required in a relevant gravitational environment to advance reliable modeling.

A typical fuel depot mission implies several operational phases: three of them are schematically reported in Fig. 1 with the associated physical phenomena that could appear. The figure is not meant to be exhaustive but is a graphical reference to highlight the main gaps in physical knowledge that need to be managed in the context of fuel depot missions. Specifically, the main highlighted phases are:

1. Ballistic phase during which the depot, full of fuel, is in its orbit. The fluid is at rest and in saturation conditions. Heat fluxes can induce pool boiling, meaning bubble growth induced by the superheated conditions of the tank wall. Tank depressurization for thermal control can induce pool cavitation, meaning bubble growth by superheated liquid in the presence of vapor germs. The control of these physical phenomena is important for the **conditioning and storage** of cryogenic fluids.
2. **Maneuvers** with the purpose of attitude control or docking during the rendezvous between the depot and the receiver tank might cause accelerations applied on the fluid resulting in sloshing phenomena. Sloshing, not only affects the trajectory and positioning of the vehicle, but promotes thermal mixing, de-stratification, condensation, and evaporation phenomena.
3. The final phase is the **transfer** of the propellant from the depot towards the receiver tank. The transfer will be affected both by temperature overheating and depressurization of the flow, which could induce convective boiling and hydrodynamic cavitation.

The gaps in physical knowledge considered here are grouped based on the operation they contribute to in a space depot system: conditioning, storage, maneuvers, and transfer. Because conditioning and storage is associated with phase change phenomena due to temperature and pressure variations, they are grouped in a single section.

### Conditioning and storage

For long-duration missions, the inevitably incoming thermal energy (e.g., due to radiation) and the different gravitational levels result in complex multiphase configurations^26^. After longer times, for example, during long-coasting phases, the liquid propellant reaches saturation conditions. Small overheats from the solid walls may lead to *pool boiling*. Small superheats due to depressurization may lead to *pool cavitation*. Both effects lead to the formation and the growth of bubbles (see Fig. 1)^49^. After long-coasting phases and prior to liquid transfer, the saturated fluid needs to be subcooled to avoid flow cavitation during transfer or re-ignition of the engine. Efficient cryocoolers are not available in the case of liquid hydrogen. Therefore, the cooling of liquid hydrogen is currently performed by pressure variation cycles^22^. This tank propellant conditioning process involves depressurization followed by re-pressurization. The depressurization phase will remove thermal energy from the saturated liquid phase (pool cavitation in Fig. 1) and evaporate a part of the liquid, which leads to superheated conditions. Nucleated bubbles might appear and grow at this phase^50^. Without buoyancy in microgravity conditions, the bubbles are expected to increase in size and remain near the wall, thus affecting the wall heat transfer^26^. Successively, a re-pressurization of the system is required for transfer operations. This re-pressurization will induce vapor condensation. Bubbles eventually shrink or collapse, which releases heat that causes a temperature of the liquid surrounding the bubbles. Vapor-accumulating regions could be created, resulting in undesired thermal conditions and producing issues in the propellant removal. Moreover, the phenomena could be coupled with boiling induced by thermal superheat^26^. These complex multiphase phenomena, including phase changes induced by pressure and temperature variations in microgravity conditions, are challenging to investigate experimentally and model^11^.

Validating different CFD and nodal tools with microgravity data is necessary to routinely design in-space cryogenic systems, especially with the fluid dynamics and heat transfer being tightly coupled as in two-phase cryogenic systems^20^. One of the main difficulties arises because of the multi-scale nature of the problem. The thermal characterization at the tank scale (meter scale) is impacted by these phase change phenomena arising at the bubble scale (milli-meter scale)^49^. Moreover, the phase change is driven by the thermal boundary layer at the interface and/or by phenomena arising in the microregion and microlayers in the contact line region, which may be of particular importance for cryogenic fluids that are perfectly wettable (nano-meter to micrometer scale). On top of that, the simulation of bubble cavitation, that is induced by a pressure drop, requires the use of a fully compressible or a suitable variable density approach and the coupling with accurate thermodynamic descriptions of both phases and of the saturation conditions at the interface.

Understanding the effect of the above-mentioned phenomena is of primary importance for tank thermal control. Studies devoted to phase change phenomena in microgravity have already been realized. Nevertheless, few of them have been devoted to phase change phenomena of cryogenics in microgravity, while most of them have been performed at terrestrial gravity conditions. In Table 5 the most relevant experimental studies regarding phase change phenomena in cryogenics and/or in microgravity have been collected, presenting briefly the kind of investigation, the gravity conditions or platform, the kind of liquid used, and with some highlights of the results and conclusions. The summary of gaps in physical knowledge and their application for conditioning and storage operations is show in Table 6.

**Table 5:** Experimental studies on thermodynamic characterization of fluid systems in microgravity

Reference	Investigation	Gravity Condition	Fluids	Highlights
Bellur et al.^119,120^	Condensation and evaporation in cryogenics with optical techniques	Ground	Hydrogen, Methane	Techniques are developed and applied for the characterization of phase change rates with changing temperature and pressure conditions.
Flash and splash^121–124^	Bubble dynamics in isothermal cavitation	Parabolic Flight	Water	Dynamic of bubbles in water cavitation has been studied in microgravity. Studies included: cavitation in drops, plasma-generated bubbles, strong collapse, shock wave formations and luminescence.
RUBI^125–127^	The experiment addresses fundamental questions about two-phase heat transfer during boiling processes	ISS	FC-72	Creation of bubbles on heated substrates by the short laser pulse. Contact line behavior on single bubbles. Multiple bubbles study. Bubble growth has been studied as: undisturbed growth, influenced by shear flow, influenced by electric field.
Source I^89^	Dual species system behavior in a compensated gravity environment	Suborbital flight	HFE-7000, Gaseous nitrogen	Free surface behavior, influence of temperature gradients on thermocapillary flows, heat transfer enhancement
Source II^88^	Single species system behavior in a compensated gravity environment	Suborbital flight	HFE-7000	Free surface behavior, the influence of evaporation on apparent contact angle

**Table 6:** Summary table of gaps in physical knowledge and their application for conditioning and storage

Physical knowledge’s gap	Application
Heat transfer coefficients with different phases (gas/liquid, liquid/solid, gas/solid), different conditions (e.g., gravity conditions, interfaces conditions), and different concurring mechanisms (e.g., natural convection, thermocapillary convection, etc.)	Thermal control, zero boil-off, mixing, system readiness for next status.
Creation of bubbles due to heat input or depressurization, with and without flow	Thermal control by means of depressurization/pressurization, zero boil-off
Critical heat flux (CHF) curve in relevant acceleration environments	Thermal control, tank filling
Mass transfer rates due to phase change	Thermal control, tank filling, autogenous pressurization
Mass transfer rates due to species concentration (effect of non-condensable gas)	Heterogeneous pressurization
Identification of interaction between the different scales in time and space of problems	Efficient, accurate and representative CFD simulations

### Maneuvers

Liquids embarked in vehicles react to external accelerations, hence maneuvers, depending on the different kinds of liquid/gas involved and the geometry of the container. Such movement is called sloshing. All sorts of maneuvers on a space depot might promote a sloshing appearance: station-keeping, rendezvous, docking, de-orbiting, spinning satellites, flat-spin transition, landing, in-orbit refueling, and other fast orbital maneuvers. In all these phases, the dynamics of the spacecraft interacts with the dynamics of the fluid: the movement of the spacecraft excites the fuel in the tanks, that exerts forces and moments on the container. The container transmits these disturbing forces/moments to the main body, in some cases causing structural damages and/or stability/performance loss. In non-isothermal systems, especially those using cryogenic propellants, the free surface movement gets thermodynamic mixing between phases. The thermodynamic mixing can cause undesired pressure fluctuations, which might disrupt the fuel feeding to the engine or create structural damages. During ballistic phases in space, when acceleration forces are reduced, surface tension forces dominate the dynamics of the liquid^51^: capillary and residual gravitational potential energies tend to minimize and the gas/liquid interface assumes the position of the minimum free surface energy^52,53^. The effect of external excitation on the dynamic of liquids in microgravity results thus different than the same liquid in normal gravity conditions (on Earth or under a constant accelerated field). Known cases of the effect of sloshing in space are reported in the literature: Hoffman et al.^54^ estimated that sloshing was the principal cause of the inefficient or apparently ineffective momentum damping observed on the occasion of the anomaly of the Near Earth Asteroid Rendezvous (NEAR) mission. Sloshing is also suspected to be the cause of the instability of the upper stage that brought the loss of the Falcon 1 mission in 2007^55^.

Sloshing mitigation can be realized by means of passive or active solutions. Passive solutions consist of baffles and bladders^56,57^ which are traditionally designed to reduce the impact of sloshing force/torques. A historical application of an anti-sloshing device is found in the ESA Automated Transfer Vehicle (ATV) project^58^. These devices have the advantage to increase the sloshing frequency and reduce the sloshing amplitude^51^. In the attitude control bandwidth, the resonance introduced by the perturbation appears at low frequencies, requiring to move the frequency of the sloshing modes to higher ranges. However, this increase of resonance frequency might fall in the spacecraft structure flexible modes having disrupting consequences for guidance and control performances^52,59^. Nevertheless, the introduction of supplementary structural elements into the design increases the system’s overall mass as well as the mission costs.

Active solutions might either act directly on the guidance and control (GNC) reference of the mission or to introduce actuators directly in correspondence with the disturbance source in order to actively damp the system. However, smoother angular velocity profiles and bigger tranquilizing times after aggressive maneuvers, respectively, cause loss of agility and available mission time window, while no active solutions are designed and tested in space applications to the author’s knowledge in order to mitigate the sloshing phenomenon.

High-fidelity CFD simulations can, to some extent, accurately represent sloshing phenomena in microgravity conditions. Nevertheless, integration of CFD tools with GNC algorithms often brings complicated analysis due to the numerical noise introduced in the system by CFD outputs^58^. Furthermore, the huge computational time taken by CFD simulations makes GNC certification (large number of validations for different scenarios, Monte Carlo analyses to cover system uncertainties) extremely challenging and incompatible with the industry’s deadlines. The widespread practice is to model sloshing phenomena as an equivalent mechanical system that acts like structural flexible modes^60^. Two simple linear models are commonly used: the mass-spring-damper model is able to represent the linear lateral sloshing; on the other hand the equivalent pendulum model has the main advantage to adapt the frequency mode in function of the acceleration. However, both these linear models have good accuracy for small amplitude liquid sloshing and axisymmetric tanks^61–64^. The biggest drawback is that these models do not take into account the dependency of the sloshing phenomena by the velocity and acceleration of the spacecraft that provokes non-negligible inertial forces/torques on the liquid^65^. More advanced studies, including non-linearities, were conducted by several authors^52,58,59,66–71^.

Most of the equivalent mechanical models^52,72–74^ present the difficulty to identify its constitutive parameters (e.g., liquid friction force and torque) by a semi-empirical approach. Pioneering work on the sloshing control are proposed by the following references:^65,75–77^.

Nevertheless, for model validation of the control approach, experiments are fundamentals. The research activity driven by Airbus DS in Bremen^70,78^ has to be considered pioneering in this direction: an experiment was conceived in order to study a way to control the sloshing of upper stages of launchers in the long-coasting phase by using a hexapode sloshing system. The main limit of these tests is that the experiment is driven in a normal gravity environment. Experiments relevant to microgravity conditions are difficult. Drop towers providing short time microgravity periods (about 9 s) and parabolic flights, having a high disturbing noise on accelerations, do not allow testing of the control strategy. The main space missions which provided a step forward in the analysis of sloshing in microgravity are reported in Table 7.

**Table 7:** Experimental studies on sloshing in microgravity

Reference	Investigation	Gravity Cond.	Fluids	Highlights
SloshSat-FLEVO (ESA)^68,128^	Launched on 12 February 2005 to study sloshing in a tank subjected to induced perturbations with a system of 12 nitrogen gas thrusters.	On-orbit GTO	Water	The mission operated for 8 days. Communication problems hampered the quality and quantity of data collected. Thanks to this mission COMFLO solver (University of Germany)^129^ was improved and validated.
SPHERES-Slosh (NASA)	Two free-flying satellites known as Synchronized Position Hold, Engage, Reorient, Experimental Satellites (SPHERES) are attached to opposite ends of a metal frame holding a plastic tank with green-colored water.	ISS	Water	The data were used to refine critical computer models to better solve launch and spacecraft fuel slosh. A synchronization problem between the SPHERES and the camera acquiring the sloshing images results in a limited validation use of the data^130^.
SPHERES thether-slosh (NASA)^131^	Experimental test-bench to study automated strategies for steering passive cargo. It consists of two automated satellites (SPHERES) connected to two different tanks: a liquid-filled tank and a solid tank of same mass.	ISS	Water	The evaluation of how the fluid and solid tanks affected the closed-loop control of the tethered system can inform future development of control strategies. The results appear to indicate the correct application of control effort to reach closed-loop set points.
FLUIDICS (ESA)^132,133^	Experimental test-bench to validate the Direct Numerical Simulation for two-phase flow with the real fluid’s behavior within a spherical tank under microgravity conditions.	ISS	Water	Excellent agreement between experimental results and simulation with DIVA^®^ (Dynamics of Interface for Vaporization and Atomization). Control of sloshing was not a goal of this mission.

A last mention is given to the interaction of sloshing with the thermodynamic system. The literature on this topic is divided into two major sets of research campaigns: ground-based and microgravity. While the ground-based campaigns cannot achieve dynamical similarity with the microgravity environment, several studies, such as references^79–84^, have built essential foundational knowledge on the physics of the problem. These campaigns characterized the thermodynamic response of partially filled tanks under different sloshing excitations, working fluids, pressurization techniques, pressurant gasses, and tank designs (e.g., with or without baffles). The state of the art on microgravity experiments has been mainly derived from drop tower experiments (5 s of microgravity)^85–87^, and sounding rocket flights (6 min of microgravity)^88,89^ campaigns. In such experiments, the gas-liquid interface retains a stable shape with a rising contact line along the walls, and periodic oscillations of its center along the axial direction. Although these conditions allow for detailed investigations of heat and mass transfer in a microgravity environment, the stable shape of the free surface does not account for more complex free surface behavior which might be encountered in long ballistic period and thruster excitations. The summary of gaps in physical knowledge and their application for maneuvers is show in Table 8.

**Table 8:** Summary table of gaps in physical knowledge and their application for maneuvers

Physical knowledge’s gap	Application
Liquid motion with free surfaces (often referred to as sloshing) at different acceleration regimes caused by any kind of excitation or environmental conditions.	GNC (long-coasting dynamic control, accurate positioning), reduce overdesign weight, tank (re)-filling, fuel transfer towards the engine.
Inertia-induced liquid jets (Geysering)	Tank filling, optimize settling maneuvers
Two-phase distribution	Mass gauging, liquid-only removal, gas-only removal, droplet injection for cooling

### Transfer

The transfer of fuel from a supply tank to a receiver tank involves several major phenomena: the chill-down of the line and the creation of different flow regimes, depending on the temperature and pressure conditions of the line, fluid-hammer effects, and depressurization of the supply tank. Starting from the situation in which the supply tank is properly conditioned, transfer can start. When the cryogenic fluid enters the pipe, evaporation occurs immediately, and the flow during the chill-down phase becomes a two-phase flow. The topology of such a flow has a strong impact on the heat transfer and the pressure drop along the channel. Therefore, generally, the first step in two-phase flow experiments, or models, is to determine the two-phase flow regime, which is dependent on many factors, such as fluid velocity, fluid density, fluid quality, gravity, and pipe size. At Earth gravity, for horizontal flow, the flow regime is visually classified as bubbly flow, plug flow, stratified flow, wavy flow, slug flow, and annular flow^90^. For vertical flow, the flow regimes include bubbly flow, slug flow, churn flow, and annular flow^91^. The only difference between the horizontal and vertical flows is the effect of gravity that causes the horizontal flow to become non-symmetrical to the tube centerline. Regimes of common two-phase flow such as air–water have been extensively mapped from experiments. However, the published data for cryogens are limited^92–94^. The influence of microgravity on the chill-down process has to be taken into account. Indeed, in microgravity the two-phase flow patterns and the heat transfer characteristics should be the same, whatever the orientation of the pipe, and the flow should be not stratified^95^. The difference in density and inertia under terrestrial conditions has an impact on the pipe flow distribution: the two phases are usually non-uniformly distributed across the pipe. The absence of gravity impacts the flow regimes, the pressure drop, and the heat transfer. In space, the effect of surface tension forces and surface phenomena is supposed to be more important than on Earth^93,96^. First modeling efforts of chill down based on one-dimensional descriptions were carried out in the sixties^97,98^. These one-dimensional models provided simple, but acceptable estimations of the chill downtime (the time required to achieve steady-state cryogenic flow). However, they neglected the transient characteristic of the chill down, the influence of the flow regimes and, in most cases, the influence of the heat transfer regimes. Last but not least, no estimation of the instantaneous wall and bulk fluid temperature was possible.

During cryogenic chill down, the pipe wall is in contact either with the liquid or with the vapor: the characterization of liquid/solid heat transfer is much more complex than the one of gas/liquid due to the occurrence of different phenomena. Indeed, when the cold liquid starts flowing in the pipe and chill-down occurs, the heat transfer regimes observed are film boiling, transition boiling (which is often neglected), nucleate boiling, and convective heat transfer. The transition from one regime to another depends on several parameters: wall heat flux, fluid properties, and wall temperature. If the wall temperature is higher than the Leidenfrost temperature, film boiling is assumed to occur; if the temperature is between the Leidenfrost temperature and the transition one, the nucleate boiling regime is considered as the actual state of the flow^99^.

Recent activities are mainly focused on the definition of chill-down flow regimes and related heat transfer characteristics, to be able to correctly describe the phenomena and to develop adequate models for prediction. A large amount of work is done on ground, but chill-down in depots occurs in microgravity conditions. Some activities include test-bench and experiments to reproduce microgravity conditions^96,100,101^. Chill-down process optimization is also a promising field of investigation, which aim at continuously improving the efficiency of cryogenic fluid applications. These two research activities are developed complementary with the common goal of reaching a comprehensive capability of chill-down management. Liquid nitrogen is often selected as a working fluid because, even if its thermophysical properties do not represent liquid hydrogen and oxygen perfectly, it is a first step towards thermodynamic similarity and an experimental cryogenic environment with respect to using classic laboratory fluids, e.g. alcohols. The scientific works related to chill-down, selected in Table 9, are oriented to applications such as cryogenic engines and propellant storage stations (used for spacecraft supply). Finally, another phenomenon of relevance in a transfer line is the so-called fluid-hammer^102–104^. The complex interaction of the flow field and liquid-vapor phase transitions, developing of boiling and condensation in the line due to pressure fluctuation, generates instabilities. The feature of such phenomena involves the slow propagation of pressure fluctuations in two-phase regions, vapor accumulated downstream at warmer pipe sections, and vapor bubbles coalescence^105–107^. Mechanical vibrations can play a role in interacting with low-frequency pressure disturbances. If the thermal-hydrodynamic disturbances generate large-amplitude flow oscillations and hydraulic shocks (fluid-hammer), a loss of thermal-flow control and mechanical damages might occur^108^. While several investigations have been performed to study cryogenic fluid-hammer in gravity conditions^109–111^, microgravity experiments are still limited^112,113^. The summary of gaps in physical knowledge and their application for fluid transfer operations is show in Table 10.

**Table 9:** Experimental studies on cryogenic chill-down

Reference	Investigation	Gravity Cond.	Fluids	Highlights
Velat^92^	Experimental investigation to collect detailed information on flow structure, flow properties, and heat transfer mechanisms associated with cryogenic chill-down.	1g	Liquid nitrogen	Visualizations of the entire chill-down process are documented among a range of mass fluxes. The existence and magnitude of circumferential and small axial temperature gradients in the transfer line during the various phases of chill-down is reported.
Hu et al.^134^	Liquid nitrogen chill-down rates and flow patterns between upward flow and downward flow in a vertical pipe.	1g	Liquid nitrogen	Increasing mass flow rate, rewetting temperature, and quench front velocity increase while the critical heat flux decreases. The total chill-down time for upward flow is longer than for downward flow. Critical heat flux, heat transfer coefficient, and the quench front velocity are higher for upward flow.
Rame and Hartwig^135^	Liquid hydrogen chill-down is experimentally studied for continuous and pulsed flow conditions	1g	Liquid nitrogen	The authors propose a connection between the non-monotonically decreasing temperature and the flow conditions, which increase the heat transfer coefficient.
Yuan et al.^93,96^	Liquid nitrogen chill-down process under both normal gravity and microgravity conditions	0g, drop tower	Liquid nitrogen	The bottom wall heat flux is lower in 0g than in 1g. Wall temperature and inlet flow rate do depend on gravity.
Kawanami et al.^101^	Liquid nitrogen forced convective boiling for low mass velocity in terrestrial and microgravity conditions	1g, 0g	Liquid nitrogen	Heat transfer and quench front velocity is 20% higher in 0g. Gravity has no effect on the maximum heat flux, which increases exponentially with the quench front velocity.
Hartwig et al.^136^	Chill-down in microgravity using pulse flow and low-thermally conductive coatings	Parabolic Flight	Liquid nitrogen	The tested combination of coatings and pulsating flow enhances significantly the performances of the chill-down: 75% reduction of mass consumption.
Sarae et al.^45^	See Table 4.			
Kinefuchi et al.^46^	See Table 4.

**Table 10:** Summary table of gaps in physical knowledge and their application for fluid transfer operations

Physical knowledge’s gap	Application
Liquid/Gas interfaces during transfer in all gravity conditions	Tank (re)-filling, fuel in transfer lines
Heat transfer coefficients with different phases (gas/liquid, liquid/solid, gas/solid), different conditions (e.g., gravity conditions, interfaces conditions), and different concurring mechanisms (e.g., natural convection, thermocapillary convection, etc.)	Tank (re)-filling, fuel in transfer lines
Creation of bubbles due to heat input or depressurization, with and without flow	Fuel in transfer lines, flow control (regulation valves, etc.), material fatigue.
Effect of gravity on the creation of bubbles (cavitation or boiling) and presence of flow	Fuel in transfer lines, flow control (regulation valves, etc.), material fatigue.

## Summary and outlook

Several nations strive to enlarge their horizon of exploration by extending their presence in space, and aiming to go back to the Moon and to reach Mars. A versatile, reliable, and efficient transportation is required to enable the presence of human beings in deep space. The most promising propulsion systems are based on cryogenic liquids and use hydrogen or methane as fuel and oxygen as oxidizer. For the development of a lunar economy and for human missions to Mars, refueling in orbit will be necessary. In this paper, we reviewed reference missions and architectures for cryogenic depots and analysed the fundamental operations of refueling in orbit, i.e., conditioning and storage, maneuvers, and transfer. We summarized the physical phenomena associated with these operations and described gaps in knowledge that need to be filled in order to enable space depots. Our review is by no means exhaustive, but aims to highlight the scientific challenges in the fields of thermodynamics, fluid dynamics, and structural mechanics, and more importantly, their nonlinear couplings, that are open. The solution to these challenges would lead to new and more capable technologies. In a nutshell, the synergies between scientific and technological exploration strategies for deep space exploration promise to open new horizons of research.
